# Regional variation in lifestyle patterns and BMI in young children: the GECKO Drenthe cohort

**DOI:** 10.1186/s12942-022-00302-7

**Published:** 2022-07-01

**Authors:** Rikstje Wiersma, Richard H. Rijnks, Gianni Bocca, H. Marike Boezen, Esther Hartman, Eva Corpeleijn

**Affiliations:** 1 Department of Epidemiology, University Medical Center Groningen, University of Groningen, PO Box 30.001, 9700 RB Groningen, The Netherlands; 2 Department of Planning, Faculty of Spatial Sciences, Urban & Regional Studies Institute, University of Groningen, Groningen, The Netherlands; 3 Department of Pediatrics, Beatrix Children’s Hospital, University Medical Center Groningen, University of Groningen, Groningen, The Netherlands; 4 Center for Human Movement Sciences, University Medical Center Groningen, University of Groningen, Section F, PO Box 196, 9700 AD Groningen, The Netherlands

**Keywords:** Childhood obesity, Diet, Sleep, Screen time, Physical activity, Sedentary time, Spatial analyses, Preschool

## Abstract

**Background:**

A better understanding of lifestyle behaviours of children < 7 years and the relation with childhood overweight is needed. The aim of our prospective study was to examine how lifestyle patterns in young children are associated with the development of childhood overweight. As ecological models suggest focusing on not only the child as an individual, but also their environment, we also considered the role of socio-economic status (SES) and spatial clustering of lifestyle and body mass index (BMI).

**Methods:**

In 1792 children (aged 3–6 years) participating in the GECKO Drenthe cohort, diet, screen time, outdoor play and sleep were assessed by questionnaires and moderate-to-vigorous physical activity and sedentary time by accelerometry (Actigraph GT3X). At 10–11 years, height and weight were measured to calculate age- and sex-specific standardized BMI z-scores (zBMI). Lifestyle patterns were identified using principal component analysis. To assess spatial clustering for the lifestyle patterns and zBMI, we calculated the Global Moran’s I statistic. Linear- and logistic regression models, taking into account SES, were performed to examine the association between the lifestyle patterns and the development of overweight. For the spatial analyses, we added spatial terms for the determinants, the outcome, and the error term.

**Results:**

Three lifestyle patterns were identified: (1) ‘high activity’, (2) ‘low screen time, high sleep and healthy diet’, and (3) ‘high outdoor play’. No associations were observed between the ‘high activity’ or ‘high outdoor play’ patterns at young age with the development of childhood overweight (all p > 0.05). In contrast, children who adhered to the ‘low screen time, high sleep and healthy diet’ pattern had lower odds to become overweight and a lower zBMI at 10–11 years (odds ratio [95% CI] = 0.766 [0.65; 0.90]). These findings remained similar after taking SES into account. Regarding the spatial analyses, we found spatial clustering of zBMI, but no spatial clustering of the lifestyle patterns.

**Conclusions:**

Low screen time, high sleep duration and a healthy diet cluster into a pattern that seems favourable in the prevention of childhood overweight, independent of individual SES. The spatial analyses suggest that there are likely other neighbourhood factors that contribute to the spatial clustering of childhood overweight.

**Supplementary Information:**

The online version contains supplementary material available at 10.1186/s12942-022-00302-7.

## Background

Childhood obesity is a growing problem within society. In 2019, almost 6% of children under the age of five worldwide were affected by overweight or obesity [1]. In the Netherlands, the percentage of children (4–12-years old) affected by overweight or obesity in 2019 was 12.0% [2]. A large global study comparing body mass index (BMI) trajectories for ages 5–19 years in 200 countries showed that the BMI in the Netherlands is close to the world median [3]. During childhood, many children with obesity develop health problems that previously emerged only in adults, such as cardiometabolic, pulmonary, and psychosocial complications, orthopaedic disorders and cancer [4, 5]. Approximately 30–50% of children with obesity will remain affected by obesity in adulthood [6, 7]. Because in young age, the acceleration of BMI is particularly high between 2 and 7 years, and overweight in childhood is likely to persist into adulthood, prevention should start at a young age [8, 9].

Known lifestyle factors that play an important role in the development of overweight and obesity are an unhealthy diet, low levels of physical activity (PA), increased sedentary time (ST), increased screen time, and a lack of sleep [10–13]. In children and adolescents of all ages, these lifestyle factors cluster in certain lifestyle patterns, depending on age, sex and socio-economic status (SES) [14]. A recent review including twenty-eight papers published between 2007 and 2019 looked at the association between lifestyle patterns and overweight in children between 5 and 12 years of age [15]. About 70% of the included studies showed significant associations between lifestyle patterns and childhood overweight [15]. However, the review included only six studies that were conducted in younger children (< 7 years), and only one prospective study included sleep as lifestyle factor [15]. For young children, little is known about the influence of lifestyle patterns on the development of overweight and obesity. In addition, studies including sleep as a lifestyle factor are scarce, despite sleep being convincingly related to overweight, potentially in interaction with other lifestyle behaviours [13, 16]. So far, one prospective and one cross-sectional study included sleep as a lifestyle factor when examining the association between lifestyle patterns and childhood overweight in children around the age of 5–7 years [17, 18]. For children of this age, adhering to a ‘low PA and high screen time’ pattern, ‘short sleep and unhealthy diet’ pattern or ‘high screen time and unhealthy diet’ pattern seemed to increase the risk of becoming affected by childhood overweight [17, 18]. To get a better understanding of how the clustering of lifestyle factors in young children is related to the development of childhood overweight, longitudinal studies in younger children are needed, taking into account multiple lifestyle factors, including diet, objectively measured PA and ST, and sleep.

In the search for opportunities for obesity prevention interventions, ecological models suggest focusing on not only the child as an individual, but also their environment. The ecological models have been designed to explain healthy or unhealthy behaviours by considering individual characteristics combined with influences of environmental and policy levels [19, 20]. Ecological models have been involved in behavioural sciences and public health, and focus on the nature of people’s transactions with their physical and sociocultural environment [21]. In children, parents have a major influence on children’s health behaviour, but school, social policies and social norms also play an important role [19]. These different levels have already been taken into account by obesity interventions based on a community approach, which involve the school environment for example [22]. To illustrate the role of parents, a study of Dutch preschoolers showed that, for example, more parks and playgrounds in the neighbourhood were associated with less ST and more moderate-to-vigorous PA (MVPA) compared to children with fewer PA facilities in their living environment [23]. This association was mediated by increased parental support for children to play sports if the parents perceived more PA facilities in their neighbourhood [23]. One way to take into account the contribution of the child’s environment to the clustering of lifestyle and the development of overweight, is by looking at the spatial clustering of children’s lifestyle patterns and BMI and by examining the effect of spatial spillovers of the child’s environment. The spatial spillovers, in our case, can be defined as the effect of neighbourhood differences in lifestyle on children's BMI's [24]. Such spatial analyses provide opportunities to tailor obesity prevention interventions for regions and high risk groups rather than individuals, which can improve the effectiveness and reach of obesity prevention programs.

The aim of our study was to examine how lifestyle patterns in young children are associated with the development of childhood overweight, taking into account children’s individual SES and spatial clustering. As a first step, we explored the spatial clustering of BMI. Secondly, we identified lifestyle patterns based on diet, screen time, outdoor play, sleep and objectively measured PA and ST and then explored the spatial clustering of these lifestyle patterns. Third, we examined the association of the lifestyle patterns with BMI and the development of childhood overweight and then studied the effect of children’s individual SES and spatial spillovers of their environment.

## Methods

### Study design and participants

The GECKO (Groningen Expert Center for Kids with Obesity) Drenthe study is a population-based birth cohort with a focus on early risk factors for overweight and obesity. Details of the GECKO Drenthe study are described elsewhere [25]. In 2006, almost three thousand pregnant women living in the Province of Drenthe, the Netherlands, were recruited. Recruitment was carried out by obstetricians, midwives and general practitioners, supported by a media campaign. Monitoring of the children started from the last trimester of the pregnancy and is still ongoing. For the current study, we used data collected on lifestyle factors between 3 and 6 years (August 2008 to April 2014) and overweight at ages 5–6 and 10–11 (August 2016 to July 2018). Children were included if they had information on at least one lifestyle factor and had data on overweight at the age of 10–11 years. Written informed consent was obtained from parents, and the study was approved by the Medical Ethics Committee of the University Medical Center Groningen in accordance to the declaration of Helsinki of 1975 as revised in 1983. The study is registered at http://www.birthcohorts.net.

**Fig. 1:**
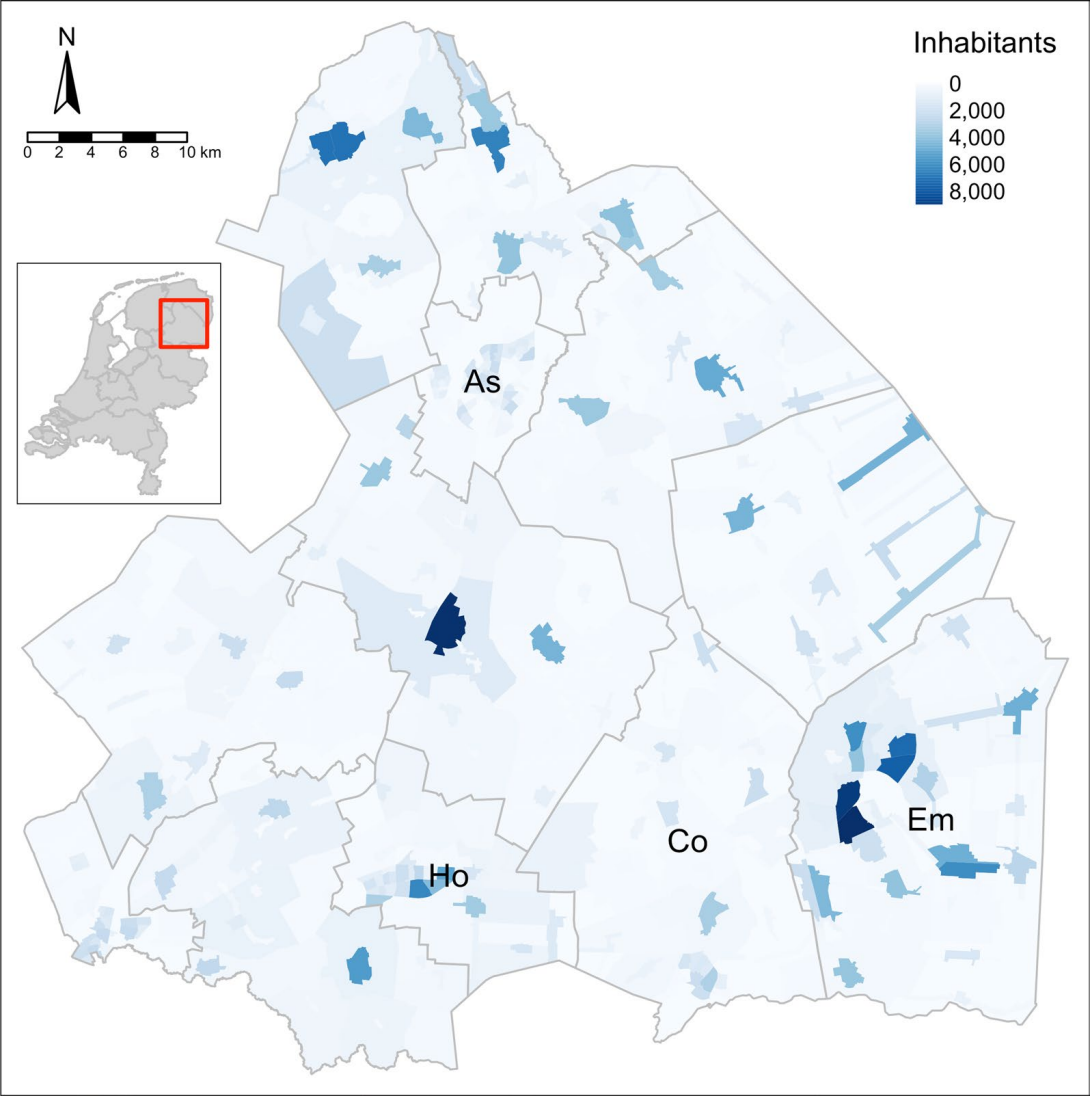
Population density in the province of Drenthe, The Netherlands. The darker the colour, the more inhabitants in that area. The four largest municipalities by inhabitants are labelled, from largest to smallest: Emmen, Assen, Hoogeveen, Coevorden

Drenthe is a province in the northern Netherlands with 12 municipalities. On January 1st 2020, the population of Drenthe was estimated at 493,682 inhabitants [26]. Drenthe is characterised by the many rural areas, and has a population density of 188 inhabitants per km^2^. Figure 1 gives an overview of the population density in the different areas of Drenthe. Within the Netherlands, Drenthe is the province with the highest prevalence of overweight (52.9% in 2016) [27].

### Lifestyle factors

We focused on the following six lifestyle factors: diet, screen time, outdoor play, sleep, physical activity and sedentary time.

#### Diet

At the age of 3–4 years, the parents of the children filled in a validated Food Frequency Questionnaire (FFQ) [28]. The FFQ includes questions about the intake of 71 food products. The parents reported if, and how often, their child on average consumed a product every week for the last month. In addition, the amount of intake and the composition was asked. Data for children for whom the FFQ was not filled in completely or the dietary intake was unreliable were excluded. The reliability of reported dietary intake was assessed using the Goldberg cutoff method and was based on the ratio of reported energy intake and basal metabolic rate [29]. This was calculated with the Schofield equation. [30]. Detailed information about the data processing can be found elsewhere [31]. To express the quality of children’s dietary pattern, diet scores were calculated based on the Lifelines Diet Score (LLDS) [32]. The LLDS ranks the relative intake of nine food groups with proven positive health effects and three food groups with proven negative health effects. For the positive food groups, that is, vegetables, fruit, whole-grain products, legumes and nuts, fish, oils and soft margarines, unsweetened dairy, coffee, and tea, higher scores are awarded to higher quintiles of consumption. For the negative food groups, that is red and processed meat, butter and hard margarines, and sugar-sweetened beverages, higher scores are awarded to lower quintiles of consumption. The young children in the current study did not drink coffee, therefore this item was not included. The scores per food group were accumulated, resulting in a diet score ranging from 0 to 44 points, with higher scores corresponding to better diet quality [31, 32].

#### Screen time and outdoor play

Data on screen time and outdoor play were collected using questionnaires when the children were about 3–4 years old. Parents filled in two questions about how many days per week and for how long their child watched television, played computer games or played outside. The time that children watched television and played computer games was added together into total screen time, calculated as the average minutes per day. Accordingly, the time that children played outside was calculated as the average minutes per day of outdoor play. More information about the answer options and data processing can be found elsewhere [13].

#### Sleep

Data on sleep were collected around the age of 5 years. Parents reported what time children usually went to bed and what time they got up. Sleep time was calculated as the average minutes per night.

#### Moderate-to-vigorous physical activity and sedentary time

PA and ST were measured objectively with tri-axial accelerometers (ActiGraph GT3X, ActiGraph, Pensacola, FL) when the children were approximately 4–6 years old. The accelerometer was placed on the child’s right hip with an elastic belt and worn during all waking hours for four consecutive days, except while bathing or swimming. Data was collected using a frequency of 30 Hz and analysed with 15 s epoch recordings. Non-wearing time was defined as periods of at least 90 min with zero counts [33]. A valid measurement was defined as having at least three days with a weartime of more than 600 min per day. More information about the data processing can be found elsewhere [34]. Minutes per day spent in MVPA (≥ 3908 cpm) or ST (≤ 819 cpm) were assessed using cut-off points developed by Butte and colleagues [35].

### Overweight

Height and weight at the age of 5–6 and 10–11 years were measured by trained Preventive Child Healthcare nurses according to standardized protocols. Weight was measured in light clothing using an electronic scale with digital reading, and recorded to the nearest 0.1 kg. Height was assessed using a stadiometer and recorded to the nearest 0.1 cm. Accordingly, BMI was calculated as weight/height^2^. BMI was transformed into age- and sex-specific standardized z-scores, using Dutch growth analyser software (Growth Analyzer 3.5; Dutch Growth Research Foundation, Rotterdam, The Netherlands) with population data from 1997 as the ref. [36]. Children were classified as affected by underweight, normal weight, overweight, or obesity using the age- and sex-specific cut-offs for children based on Cole et al. [37].

### Confounding variables

For SES, a recently developed indicator for standardized household income, the Equivalized Household Income Indicator (EHII), was used. This cohort-specific household income indicator is specifically developed for European birth cohort studies [38]. The household disposable income is potentially one of the most important single indicators of SES, as it is a direct measure of material resources [38]. External data from the pan-European Union Statistics on Income and Living Conditions (EUSILC) surveys and data from the GECKO cohort were used. Within the GECKO cohort, the following predictors for the EHII were available: parental age, education level, occupational status and country of birth, cohabitation status (living with/without a partner), dwelling type and family size. A prediction model was constructed using EUSILC data of the Netherlands from 2011 and validated with data from 2015. The prediction model, resulting in regression coefficients needed to derive the EHII, had a good overall performance (R^2^ = 0.455). The currency of the EHII was EURO. As income is not a linear variable, the EHII was scaled (mean = 0, standard deviation = 1). The predictors used to estimate the EHII and smoking during pregnancy were self-reported by the parents when the child was born. For descriptive purposes, maternal education level was divided in the following three groups: (1) no education—lower general secondary education, (2) senior secondary vocational education—higher general secondary education/pre-university education, and (3) higher vocational education—university.

### Geographical information

Addresses of the participants around the age of 5–6 were used. The addresses included are postcodes (four digits and two letters), house numbers, and house number sub-specification (e.g., a, b, or − 1, − 2) where relevant. We combined this data with the Dutch Building Registry (Basisadministratie Gebouwen, BAG) [39]. From the BAG, we obtained all residential properties in Drenthe, their xy-coordinates (projected in Amersfoort EPSG:28992, unit of distance: metres), and addresses. We found a one-to-one match with 2815 out of 2906 observations with address information (96.9%).

For completeness, we approximately matched the remainder of the dataset based on their six digit postcodes. To do so, we calculated the centroid (median) xy-coordinate for all residential properties in each respective six-digit postcode based on the BAG dataset, and assigned this to the observations.

### Statistical analysis

All statistical analyses were performed using R version 4.0 and IBM SPSS Statistics version 23. The R-packages used for the analyses were *tidyverse*, *tmap*, *sf*, *kableExtra*, *spatialreg*, *GWmodel*, *mice*, *osmdata*, *raster*, and sp. Differences in lifestyle, overweight and maternal education level between tertiles of the EHII were assessed using One-Way ANOVA tests and χ^2^-tests.

#### Multiple imputation

The data on lifestyle factors and overweight were collected at different time points, which has resulted in missing data. Most data were missing for practical reasons, mainly due to logistical problems with the distribution of the questionnaires. Therefore, we assumed that the data was missing at random. For the multiple imputation the *mice* package was used [40]. The minimum required number of imputed datasets was based on the missing data rate and calculated as the number of cases with missing data divided by the total number of cases. A separate univariate imputation model was specified for each variable. Bayesian linear regression (norm) and predictive mean matching (pmm) were used for normally and non-normally distributed continuous data, respectively. For categorical variables, proportional odds models (polr) were used for ordered data and polytomous logistic regression (polyreg) for unordered data. Logistic regression (logreg) was used for binomial data. We created a predictor matrix to specify which variables should be used as predictors in which imputation model. Variables were ordered according to the amount of missing data. Convergence was monitored by visual diagnosis and by comparing the observed and imputed values using independent t-tests.

#### Principal component analysis

To identify different lifestyle patterns, principal component analysis (PCA) was performed using IBM SPSS Statistics version 23. The lifestyle factors were scaled before the analysis. Within the PCA, oblique rotation was used because the lifestyle factors are likely to correlate. The number of components was determined based on visual inspection of the scree plot and an eigenvalue > 1. The scores for each lifestyle pattern were calculated by summing the six lifestyle factors weighted by their factor loadings. PCA was performed in each imputed dataset separately. Subsequently, factor loadings were pooled using Generalized Procrustes Analysis [41]. The overall explained variance was estimated by taking the mean of the percentages of explained variance in each imputed dataset.

#### Spatial clustering

To get a visual impression of the spatial clustering of children’s lifestyle patterns and overweight without compromising the anonymity of the participants, geographically weighted summary statistics were calculated using *GWmodel* and plotted [42, 43]. The geographically weighted statistics were calculated for a grid imposed on the province of Drenthe (cell size 1000 m with an adaptive bandwidth of 20 neighbours). The distribution of the geographically weighted mean and the local variation (geographically weighted standard deviation) in the province of Drenthe are shown. To further explore the spatial clustering of the lifestyle patterns and overweight the Global Moran’s I statistic was calculated using *spdep* [44, 45]. The Global Moran’s I returns the probability of finding the observed spatial distribution of values (e.g., zBMI) under the assumption of a random spatial distribution. The Global Moran’s I was calculated for both the lifestyle patterns and zBMI at 10–11 years. The level set for significance was p < 0.05.

#### Regression models and spatial analysis

Linear regression models were performed to examine the association between the lifestyle patterns and overweight. Outcomes for overweight included overweight or obesity at 10–11 years (yes/no) and zBMI at 10–11 years. In regression model 1 we adjusted for sex, age, smoking during pregnancy, accelerometer weartime, energy intake and zBMI at 5–6 years. In model 2, we studied the influence of SES by adding standardized EHII as a confounder. If SES was significant, an interaction term for SES and lifestyle was added. Subsequently, spatial analyses were performed for the continuous outcome zBMI at 10–11 years.

The spatial analyses were initiated by adding spatial terms to model 2 following a conventional spatial econometric framework. A detailed description of the spatial analysis can be found in Additional file 1. In short, we followed convention by first estimating a general nesting spatial model (for a full description of the eight possible models see Halleck-Vega and Elhorst [24]. This model includes spatial autocorrelation of the dependent variable (neighbours’ zBMI at 10–11 years affect zBMI at 10–11 years), spatial spillovers of the independent variables (neighbours’ lifestyles affect zBMI at 10–11 years), and spatial autocorrelation of the error term (corrects for errors that are not independent and identically distributed). We subsequently excluded insignificant spatial terms and fit a spatial autoregressive combined model, a spatial durbin error model and spatial error model. For this study, the spatial weights matrix was constructed using the 10 nearest neighbours (where neighbours refer to participants) to ensure an equal number of neighbours for each individual (more detail on the structure of the spatial weights matrix can be found in Additional file 1). All regression models were performed using *spatialreg* in each imputed dataset separately, and combined using Rubin’s rules [44–46]. As sensitivity analyses, we also performed the analyses with one pattern at a time and performed the analyses on the subset of children with complete information. Spatial regressions were performed for the dependent variable zBMI at 10–11 years. At present there is no equivalent maximum likelihood method for imputed data for these regressions with a binary outcome variable. Therefore, it was not possible to perform the spatial analyses for the dichotomous outcome overweight or obesity.

## Results

A total of 2102 children had data on at least one lifestyle factor and 2198 children had data on overweight at 10–11 years. In total, 1818 children had data on at least one lifestyle factor and overweight at the age of 10–11 years. The percentage of missing values across the six lifestyle factors varied between 24.5% for sleep and 54.6% for screen time (see Additional file 2). In total, 1519 out of 1818 (84%) children had at least one missing observation, therefore 84 imputed datasets were created. The geographical location of twenty-six children was unknown, resulting in 1792 children included in the analyses. The descriptive characteristics of the study participants are shown in Table 1. Children from a lower SES had a lower diet score and spent less time sedentary compared to children from a higher SES. In addition, BMI and the prevalence of overweight and obesity was higher in children with a low SES.

**Table 1:** Descriptive characteristics of the study sample

	Socio-economic status^a^		
	Low (756–1773 Euro)	Middle (1773–2233 Euro)	High (2233–2943 Euro)
Sex (%boys)	285 (49.9%)	298 (52.1%)	276 (48.3%)
**Lifestyle (3–6 years)**			
Diet	19.8 ± 6.2	21.4 ± 6.1^c^	22.2 ± 5.9^c^
Outdoor play (min/day)	90.0 [17.0–180.0]	90.0 [20.3–180.0]	81.0 [20.3–180.0]
Screen time (min/day)	63.0 [8.0–180.0]	56.3 [8.3–139.5]	49.9 [9.1–135.0]
Sleep (min/day)	689.2 ± 27.6	688.5 ± 29.4	685.5 ± 28.5
MVPA (min/day)	66.1 ± 24.3	65.6 ± 25.0	62.2 ± 23.4
Sedentary time (min/day)	365.3 ± 51.6	372.6 ± 56.7	379.4 ± 55.0*
**Overweight (5–6 years)**			
BMI (kg/m^2^)	16.2 ± 1.6	16.0 ± 1.5	15.9 ± 1.3*
zBMI	0.32 ± 0.84	0.25 ± 0.84	0.19 ± 0.78*
* Weight status*			
Underweight	25 (4.7%)	28 (5.5%)	36 (6.8%)
Normal weight	447 (84.3%)	433 (84.2%)	449 (84.6%)
Overweight	48 (9.1%)	42 (8.2%)	41 (7.7%)
Obesity	10 (1.9%)	11 (2.1%)	5 (0.9%)
**Overweight (10–11 years)**			
BMI (kg/m^2^)	18.2 ± 3.0	17.9 ± 2.8	17.4 ± 2.4^c^
zBMI	0.35 ± 1.1	0.25 ± 1.1	0.11 ± 1.0^c^
* Weight status*^c^			
Underweight	64 (11.2%)	70 (12.2%)	72 (12.6%)
Normal weight	395 (69.2%)	389 (68.0%)	431 (75.5%)
Overweight	85 (14.9%)	96 (16.8%)	55 (9.6%)
Obesity	27 (4.7%)	17 (3.0%)	13 (2.3%)
**Socio-economic status**			
EHII (Euro)	1525.9 ± 178.0	1985.4 ± 139.0^c^	2457.4 ± 172.0^c^
* Maternal education level*^b,c^			
Low	111 (19.7%)	24 (4.5%)	0 (0%)
Middle	335 (59.5%)	242 (44.8%)	181 (37.0%)
High	117 (20.8%)	274 (50.7%)	308 (63.0%)
**Moved between measurements**			
Moved (% yes)	101 (17.0%)	87 (14.5%)	96 (16.0%)

MVPA, moderate-to-vigorous physical activity. Data are presented as mean ± SD, N(%) or median [95%CI]

^a^Based on tertiles of the Equivalized Household Income Indicator (EHII)

^b^low, no education–lower general secondary education; middle, senior secondary vocational education–higher general secondary education/pre-university education; high, higher vocational education–university

^c^Significant difference between groups of socio-economic status, with the low socio-economic status group as the reference

### Spatial clustering of body mass index

**Fig. 2:**
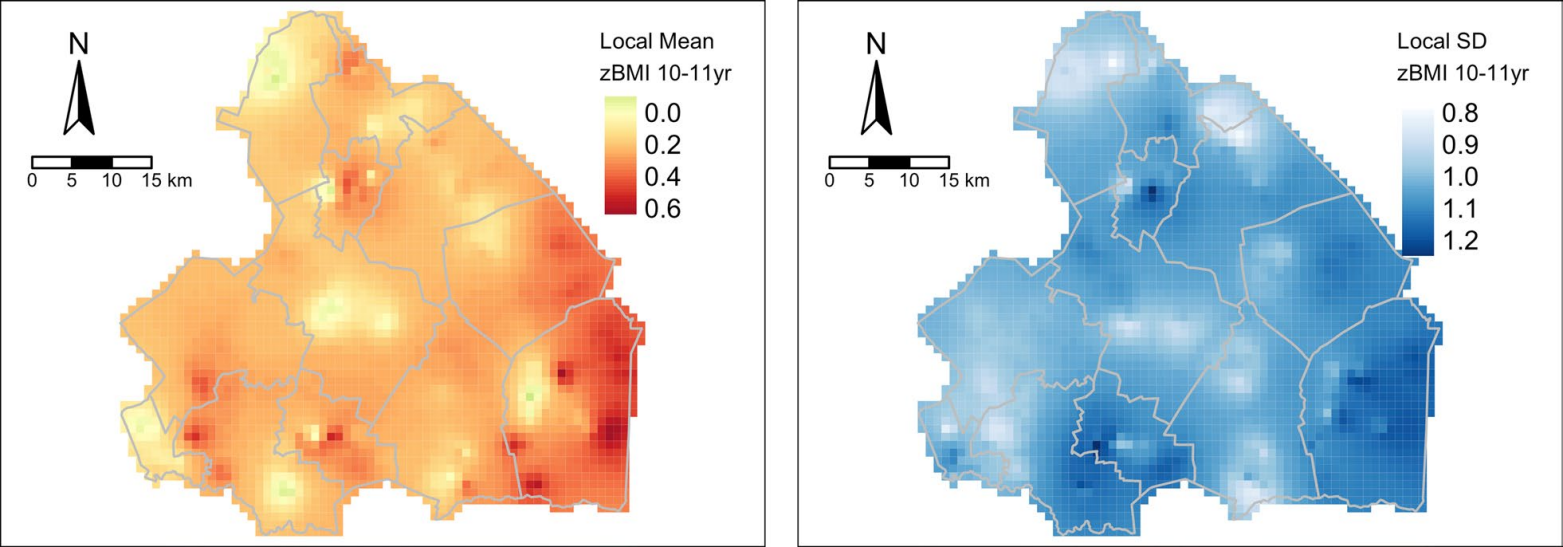
Spatial clustering of zBMI in 10–11 years old children in the province of Drenthe, The Netherlands. The left panel shows the distribution of mean zBMI (local mean) in the province of Drenthe, The Netherlands. The darker the colour, the higher the mean zBMI within that area. The right panel shows the geographically weighted standard deviation in zBMI (local SD) in the province of Drenthe, The Netherlands. The darker the colour, the higher the variation in observed zBMI in that area

Figure 2 shows the spatial clustering of zBMI at age 10–11 years. The mean zBMI was 0.23, ranging from − 3.12 to 3.29. Except for a few small areas, zBMI was relatively high in all of the Drenthe province, with the highest levels in the former peat excavation areas in the south-east of the province. The left panel in Fig. 2 highlights the elevated local mean of BMI at 10–11 years in the region, and the right panel in Fig. 2 shows that this area also has a high local standard deviation. This indicates that the BMI at 10–11 years varies more among children in this region than, for instance, the north of the province. These findings were further confirmed by the Global Moran’s I statistic, which revealed spatial clustering of zBMI at 10–11 years (0.031 ± 0.010, *p* < *0.001*).

### Lifestyle patterns and spatial clustering

**Fig. 3:**
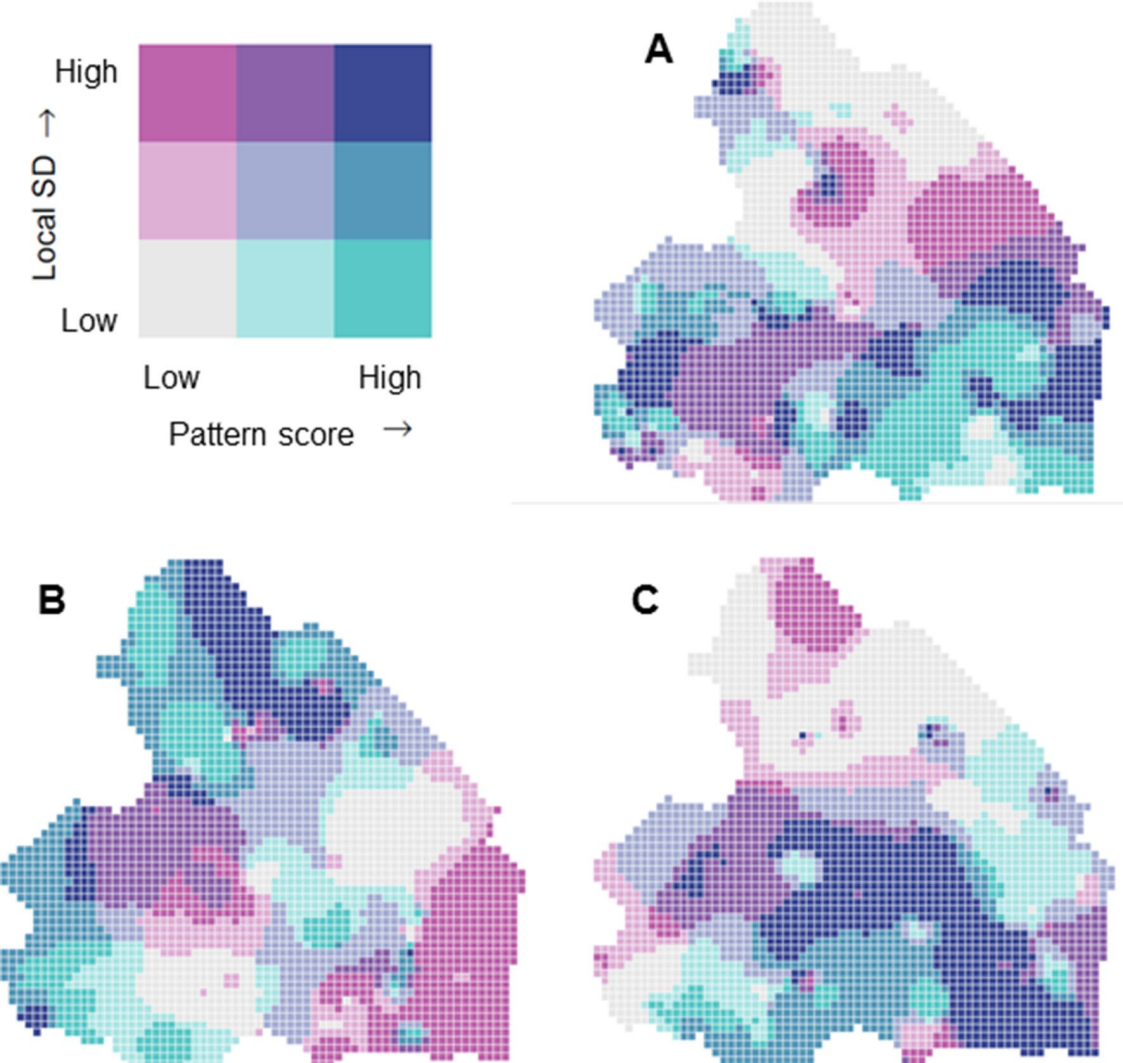
Spatial clustering of children’s lifestyle at 3–6 years in the province of Drenthe, The Netherlands. **A** ‘high activity’ pattern; **B** ‘low screen time, high sleep and healthy diet’ pattern; **C** ‘high outdoor play’ pattern. The geographically weighted mean score for each pattern was plotted against the geographically weighted standard deviation (SD) as a reflection of differences between areas (turquoise) and between children within areas (magenta). The colour palettes reflect tertiles in each variable [56]. In the grey areas (low pattern score) and turquoise areas (high pattern score), the SD is low, indicating little variation between children within the neighbourhood. In the dark blue areas, the pattern scores are high with a high SD, indicating that in that area children generally score high, but the pattern scores differ largely between children. Lastly, in the magenta areas, the mean pattern scores are low, but the variation between children is large

PCA revealed three components based on the scree plot and eigenvalues. The following lifestyle patterns were identified: (1) ‘high activity’, (2) ‘low screen time, high sleep and healthy diet’, and (3) ‘high outdoor play’ (Table 2). Overall, these patterns explained 64.8% of the variation in lifestyle. The spatial clustering of children’s lifestyle patterns is shown in Fig. 3. For each lifestyle pattern, the geographically weighted mean pattern score was plotted against the geographically weighted standard deviation of the pattern score. The Global Moran’s I statistics revealed no significant spatial clustering of lifestyle (all p > 0.05).

**Table 2:** Factor loadings and lifestyle patterns

Lifestyle factor	‘High activity’ pattern	^‘^Low screen time, high sleep and healthy diet’ pattern	‘High outdoor play’ pattern
MVPA	0.88	− 0.09	− 0.06
Sedentary time	− 0.88	− 0.06	0.04
Screen time	− 0.04	− 0.73	0.02
Sleep	− 0.09	0.51	0.26
Diet	0.03	0.61	− 0.40
Outdoor play	0.17	0.16	0.76

Explained variance	26.9%	21.2%	16.7%

MVPA, moderate-to-vigorous physical activity

Factor loadings were derived from principal component analysis. The score for each lifestyle pattern was calculated by summing the six lifestyle factors weighted by their factor loadings

### Lifestyle and the development of overweight or obesity

The associations of the lifestyle patterns with overweight or obesity at 10–11 years are shown in Table 3. The ‘high activity’ pattern and ‘high outdoor play’ pattern were not related to overweight or obesity at 10–11 years. In contrast, children with higher scores on the ‘low screen time, high sleep and healthy diet’ pattern had lower odds to become affected by overweight or obesity at 10–11 years (OR [95% CI] = 0.776 [0.65; 0.90]). These findings remained similar after taking SES into account. The association between the ‘low screen time, high sleep and healthy diet’ pattern and overweight did not differ by SES, as no significant interaction between the pattern and SES was found (OR [95% CI] = 0.959 [0.82; 1.12]). The analyses with one pattern at a time and the analyses with the subset of children with complete information showed similar estimates.

**Table 3:** Prospective associations of the lifestyle patterns with overweight or obesity at 10–11 years

	Model 1^a^		Model 2^b^	
	Odds ratio	95% CI	Odds ratio	95% CI
**Lifestyle patterns**				
‘High activity’	1.028	(0.91; 1.16)	1.017	(0.90; 1.15)
‘Low screen time, high sleep and healthy diet’	**0.766**	**(0.65; 0.90)**	**0.776**	**(0.66; 0.92)**
‘High outdoor play’	1.151	(0.91; 1.46)	1.126	(0.89; 1.43)
**Socio-economic status**				
Equivalized household income indicator	−	−	**0.829**	**(0.70; 0.98)**

^a^Model 1: analyses were adjusted for age, sex, weartime, energy intake, smoking during pregnancy and standardized body mass index (zBMI) at 5–6 years

^b^Model 2: Model 1 + Equivalized Household Income Indicator

Table 4 shows the associations between the lifestyle patterns with zBMI, taking into accout SES and spatial clustering. The ‘high activity’ pattern and ‘high outdoor play’ pattern were not related to zBMI at 10–11 years, but the ‘low screen time, high sleep and healthy diet’ pattern was. Children adhering to this pattern had a lower zBMI at 10–11 years. Similar results were found if SES was taken into account. No significant interaction between the ‘low screen time, high sleep and healthy diet’ pattern and SES was found (B [95% CI] = 0.012 [− 0.02; 0.04]). For comparison, the unadjusted estimates of the association between the lifestyle patterns and zBMI have been added in Additional file 3. With regard to the spatial analyses, the progression from the general nesting spatial model (model 4, Table 4), the spatial autoregressive combined and spatial durbin error models (see Additional file 3), to the spatial error model (model 5, Table 4) showed no evidence of spatial autocorrelation of the independent variables or spatial spillovers of the dependent variables. The spatial durbin error and spatial error model, however, did show a significant effect for the spatial error term. This indicates that spatial clustering of zBMI at 10–11 years is in part a reflection of unobserved spatial processes, independent of children’s lifestyle.

**Table 4:** Prospective associations of the lifestyle patterns with standardized body mass index at 10–11 years

	Model 1^a^		Model 2^b^		Model 3^c^		Model 4^d^	
	B	95% CI	B	95% CI	B	95% CI	B	95% CI
**Lifestyle patterns**								
‘High activity’	0.005	(− 0.02; 0.03)	0.002	(− 0.03; 0.03)	0.003	(− 0.03; 0.03)	0.002	(− 0.03; 0.03)
‘Low screen time, high sleep and healthy diet’	**− 0.076**	**(− 0.11; − 0.04)**	**− 0.072**	**(− 0.11; − 0.04)**	**− 0.071**	**(− 0.11; − 0.03)**	**− 0.071**	**(− 0.11; − 0.03)**
‘High outdoor play’	0.036	(− 0.01; 0.09)	0.031	(− 0.02; 0.08)	0.030	(− 0.02; 0.08)	0.031	(− 0.02; 0.08)
**Socio-economic status**								
Equivalized household income indicator	–	–	**− 0.054**	**(− 0.09; − 0.02)**	**− 0.050**	**(− 0.09; − 0.02)**	**− 0.052**	**(− 0.09; − 0.02)**
**Spatial components**								
Rho (spatial term for zBMI 10–11 years)	–	–	–	–	0.004	(− 0.84; 0.84)	–	–
lag_SES	–	–	–	–	− 0.054	(− 0.18; 0.07)	–	–
lag_’high activity’	–	–	–	–	− 0.021	(− 0.11; 0.07)	–	–
lag_’low screen time, high sleep and healthy diet’	–	–	–	–	− 0.022	(− 0.14; 0.10)	–	–
lag_’high outdoor play’	–	–	–	–	− 0.034	(− 0.20; 0.13)	–	–
Lambda (spatial term for error)	–	–	–	–	0.052	(− 0.78; 0.89)	**0.121**	**(0.02; 0.23)**

Additional models were run including a dummy for moving, and moving to healthier/less healthy neighbourhoods (determined at age 5 using geographically weighted mean of zBMI 5 years, converted to tertiles). Neither variable was significantly associated with standardized body mass index at 10–11 years. Results available upon request

^a^Model 1: analyses were adjusted for age, sex, weartime, energy intake, smoking during pregnancy and standardized body mass index (zBMI) at 5–6 years

^b^Model 2: Model 1 + Equivalized Household Income Indicator

^c^Model 3: Model 2 + spatial terms for the determinants, the outcome and the error term, and additional adjustment with a spatial term for zBMI at 5–6 years

^d^Model 4: Model 2 + spatial term for the error

## Discussion

The aim of our study was to examine how lifestyle patterns in young children are associated with the development of childhood overweight, taking into account children’s individual SES and spatial clustering of lifestyle and BMI, as a reflection of the influence of a broader SES environment. Three lifestyle patterns were identified: (1) ‘high activity’ pattern, (2) ‘low screen time, high sleep and healthy diet’ pattern, and (3) ‘high outdoor play’ pattern. Children adhering to the ‘low screen time, high sleep and healthy diet’ pattern had lower odds to become overweight and had a lower zBMI at 10–11 years. The spatial analyses revealed spatial clustering in zBMI, but we were not able to show significant spatial clustering of the lifestyle patterns and thus, no spatial component in the relationship between lifestyle and BMI.

The ‘low screen time, high sleep and healthy diet’ pattern seems favourable in the prevention of childhood overweight. Children with higher scores on this pattern were less likely to become affected by overweight or obesity at 10–11 years and had a lower zBMI at 10–11 years. In comparison, one prospective study in 5-years-old Dutch children examined activity-related behaviours and eating routines [47]. They found that a ‘high television time, unhealthy eating routines and increased sedentary time’ pattern was associated with childhood overweight [47]. Children adhering to this pattern showed a higher BMI and a positive trend for being overweight at 8 years of age [47]. In addition, a ‘high sports participation and a high computer use’ pattern was positively associated with an increased risk of becoming overweight at the age of 7 years [47]. The ‘high television time, unhealthy eating routines and increased sedentary time’ pattern resembles the ‘low screen time, high sleep and healthy diet’ pattern we found, accumulating the evidence that in young children screen time and diet cluster in a pattern associated with childhood obesity. In our study, we included sleep as an additional lifestyle factor and sleep also appears to contribute to this obesity-related pattern. Two previous studies in young children included sleep as well [17, 18]. In 2013, one prospective study in 6–7-years-old Australian children showed that children adhering to a ‘low PA and high screen time’ pattern or ‘short sleep and unhealthy diet’ pattern had an increased risk of becoming affected by childhood obesity compared with children adhering to a ‘healthy’ pattern [17]. More recently a cross-sectional study in 5-year-old French children showed that girls who adhered to a ‘very high television exposure and high outdoor PA’ pattern had a higher percentage of body fat compared to their peers, but no association between lifestyle and adiposity was found for boys [18]. In our study, no effects of the ‘high activity’ pattern or the ‘high outdoor play’ pattern on the development of overweight or obesity were observed. This may be surprising, however, especially in studies using objective measures to assess PA, evidence is accumulating that the influence of PA at a young age on the development of overweight or obesity is limited and that PA may become more important in middle or late childhood [48]. With regard to the lifestyle patterns, it is important to note that these can vary for example according to country, SES, child age and sex [14, 49]. Also, some lifestyle factors may cluster strongly, like screen time and eating habits, whereas others are less consistent, like outdoor play (in our data) or sports participation [18]. For example, in the ‘outdoor play’ pattern, diet and sleep had a reasonable factor loading as well (− 0.40 and 0.26, respectively), showing that more outdoor play was also related to a slightly more unhealthy diet and a bit more sleep. Another remark regarding the lifestyle patterns is that it may be surprising that outdoor play does not cluster in the ‘high activity’ pattern. Outdoor play can broadly range from sitting outside to running. In our data, outdoor play is associated with PA and other lifestyle factors, but the correlations are generally weak (and weaker for vigorous PA than for light PA, for example). Taken together, little information from prospective studies in young children is available, but lifestyle patterns characterised by a clustering of screen time and diet is related to childhood overweight. In our study, this lifestyle pattern consisted of low screen time, high sleep duration and a relatively healthy diet.

Our study demonstrated that children’s individual SES matters in the development of childhood overweight, but in the prevention of childhood overweight it may be better to target preschool children based on their lifestyle instead of targeting children by SES. SES is a broad concept, which is also reflected in the many predictors used to estimate the EHII. Although we used this comprehensive measure of SES, instead of using, for example, maternal education level, the protective effect of low screen time, high sleep duration and healthy diet on the development of overweight seems stronger than the effect of children’s individual SES. Adding the EHII to the regression models did not cause large changes in the effect estimates of the lifestyle patterns and the estimate of the EHII was smaller than the effect estimates of the ‘low screen time, high sleep and healthy diet’ pattern on childhood overweight and BMI. To add, even though single lifestyle factors differed by individual SES, the influence of the ‘low screen time, high sleep and healthy diet’ pattern on the development of overweight did not differ by individual SES. The effect of SES may be more evident in older children, as the socio-economic inequalities in childhood BMI may become larger when children grow older. Based on our results, we suggest that, at least in preschool children, it might be more useful to target obesity prevention interventions by screening for unhealthy lifestyle patterns and high BMI rather than focussing on children from low SES. Future intervention studies should examine whether targeting preschool children based on their lifestyle is indeed effective in the prevention of childhood overweight, and whether this holds for all child ages, from infancy to adolescence.

The positive association of the ‘low screen time, high sleep, and healthy diet’ pattern on the development of childhood overweight does not appear to have a regional component. Nevertheless, the spatial analyses revealed a number of interesting patterns in the data. First, zBMI at 10–11 years was significantly clustered throughout the study area, whereas the lifestyle patterns were not. Second, there were no spatial spillovers for the lifestyle patterns to zBMI, indicating that neighbourhood lifestyles do not affect BMI directly. Third, we found no evidence of spatial autocorrelation in zBMI at 10–11 years. This means that higher local BMI’s do not affect individual BMI’s. The spatial clustering of zBMI, observed in the Global Moran’s I statistic, is therefore accounted for by individual variables or other neighbourhood factors. Finally, the spatial clustering of zBMI at 10–11 years was independent of children’s lifestyle and SES, after accounting for the individual lifestyle patterns. This suggests that there are likely neighbourhood factors other than children’s lifestyle that contribute to the spatial clustering of BMI. Neighbourhood factors known to contribute to spatial clustering of obesity in the general population are urban layout and sprawl, healthy food and exercise access and the neighbourhood social environment [50]. In addition, differences in school- and municipal policies may lead to spatial clustering. Nevertheless, these neighbourhood factors would be reflected in spatial clustering of children’s lifestyles. More healthy food and exercise access could lead to more healthy diets and increased PA. Possibly, an effect of children’s lifestyle on the spatial clustering of BMI could have been found with a larger sample size, as that makes it possible to look at smaller geographical areas. In our study, the heterogeneity in lifestyle within neighbourhoods could be higher than between neighbourhoods due to the relatively large geographical areas, which could explain why we did not observe significant spatial clustering of lifestyle. Still, a detailed screening of area specific lifestyle patterns and BMI may aid in targeting obesity prevention interventions. To illustrate, the highest levels of zBMI at 10–11 years were observed in the former peat excavation areas in the south-east of the Drenthe province. These areas are known for a clustering of health and social issues [51]. Previous studies have identified a clustering of poor dietary patterns [52], overweight in adults [53], and low kidney function in the region [54]. Figure 3 shows how different former peat excavation areas may be in need of different interventions and which areas may benefit most from a certain intervention. The magenta areas in the southeast of Drenthe, for example, indicate that there is plenty of room for improvement, as the mean pattern score is low in these areas, but the standard deviation is high—an indication that there is also a large group of children who live healthily, suggesting a targeted approach or a ‘best example’ approach. In the grey areas in the southeast, there is less variation and children live more consistently unhealthy—an indication that a broader regional program may be suitable. This innovative approach can be promising for future research and policymaking. With such a regional approach, interventions can be targeted more specifically to the children in need, which allows greater profit and probably less costs.

Strengths of this study include the interdisciplinary approach, the 5-year follow up, the broad range of lifestyle factors and the objective measures for PA and ST. The study also has limitations. First, the lifestyle factors were not all measured at the same age, e.g., diet was measured at a younger age than sleep. Second, the different time points also resulted in missing data which we addressed using multiple imputations. The estimates in our study could therefore be underestimated, as the variation increased by creating multiple imputed datasets. However, we do not expect a large influence of the multiple imputations on our results, as the sensitivity analyses on the subset of children with complete information showed similar estimates. Third, we did not include variables regarding the personal physical environment of the children. However, this has been investigated previously in relation to physical activity and outdoor play in young Dutch children [23], and we do not expect a direct effect of these parameters on diet or sleep duration. Fourth, we showed that young children’s lifestyle is related to the development of overweight and we observed spatial clustering of BMI, but we were not able to show significant spatial clustering of lifestyle. Further exploration is needed to explain why there is an association between lifestyle and BMI at the individual level but not at the neighbourhood level. Despite these limitations, our study adds to the importance of a healthy lifestyle at a young age in the prevention of childhood obesity. With the mixed methods design we explored an innovative and interdisciplinary approach, which, together with the long follow-up time, provides a broad view on the relationship between children’s lifestyle and the development of childhood overweight.

## Conclusion and future perspectives

In the prevention of childhood overweight, a combination of low screen time, high sleep duration and healthy diet seems most favourable. Despite spatial clustering in childhood BMI, we were not able to show spatial clustering of children’s lifestyle patterns. We recommend additional studies with greater number of participants and smaller geographical areas, with detailed lifestyle and anthropometry. In addition, we recommend taking other factors into account, such as other behavioural factors, mental health aspects and (epi)genetics, considering interactions between these different domains [55]. Lastly, regional childhood obesity interventions targeting children in need based on their lifestyle pattern are needed to examine whether this approach is effective in the prevention of childhood overweight.

## Supplementary Information

**Additional file 1. **Detailed description of the spatial analysis.

**Additional file 2. **Description of missing data.

**Additional file 3. **Additional regression models for the prospective associations of the lifestyle patterns with standardized body mass index at 10–11 years.

## Data Availability

The datasets used and/or analysed during the current study are available from the corresponding author on reasonable request.

## Abbreviations

BMI: Body mass index
EHII: Equivalized household income indicator
EUSILC: European Union Statistics on Income and Living Conditions
FFQ: Food frequency questionnaire
GECKO: Groningen Expert Center for Kids with Obesity
MVPA: Moderate-to-vigorous physical activity
OR: Odds ratio
PA: Physical activity
PCA: Principal component analysis
SES: Socio-economic status
ST: Sedentary time
zBMI: Standardized body mass index

## References

[1] United Nations Children’s Fund (UNICEF), World Health Organization, International Bank for Reconstruction and Development/The World Bank. Levels and trends in child malnutrition: key findings of the 2020 edition of the Joint Child Malnutrition Estimates. 2020.

[2] Centraal Bureau voor de Statistiek (CBS). Lengte en gewicht van personen, ondergewicht en overgewicht; vanaf 1981. 2020.

[3] Rodriguez-Martinez A, Zhou B, Sophiea MK, Bentham J, Paciorek CJ, Iurilli ML (2020). Height and body-mass index trajectories of school-aged children and adolescents from 1985 to 2019 in 200 countries and territories: a pooled analysis of 2181 population-based studies with 65 million participants. Lancet.

[4] Gurnani M, Birken C, Hamilton J (2015). Childhood obesity: causes, consequences, and management. Pediatr Clin North Am.

[5] Pandita A, Sharma D, Pandita D, Pawar S, Tariq M, Kaul A (2016). Childhood obesity: prevention is better than cure. Diab Metab Syndr Obes.

[6] Monteiro POA, Victora CG (2005). Rapid growth in infancy and childhood and obesity in later life—a systematic review. Obes Rev.

[7] Serdula MK, Ivery D, Coates RJ, Freedman DS, Williamson DF, Byers T (1993). Do obese children become obese adults? A review of the literature. Prev Med.

[8] Geserick M, Vogel M, Gausche R, Lipek T, Spielau U, Keller E (2018). Acceleration of BMI in early childhood and risk of sustained obesity. N Engl J Med.

[9] De Kroon MLA, Renders CM, Van Wouwe JP, Van Buuren S, Hirasing RA (2010). The Terneuzen Birth Cohort: BMI changes between 2 and 6 years correlate strongest with adult overweight. PLoS ONE.

[10] Spiegelman BM, Flier JS (2001). Obesity and the regulation of energy balance. Cell.

[11] Bauman A, Allman-Farinelli M, Huxley R, James WPT (2008). Leisure-time physical activity alone may not be a sufficient public health approach to prevent obesity—a focus on China. Obes Rev.

[12] Patel SR, Hu FB (2008). Short sleep duration and weight gain: a systematic review. Obesity.

[13] Sijtsma A, Koller M, Sauer PJJ, Corpeleijn E (2015). Television, sleep, outdoor play and BMI in young children: the GECKO Drenthe cohort. Eur J Pediatr.

[14] Leech RM, McNaughton SA, Timperio A (2014). The clustering of diet, physical activity and sedentary behavior in children and adolescents: a review. Int J Behav Nutr Phys Act.

[15] D’Souza NJ, Kuswara K, Zheng M, Leech R, Downing KL, Lioret S (2020). A systematic review of lifestyle patterns and their association with adiposity in children aged 5–12 years. Obes Rev.

[16] Fatima Y, Doi SAR, Mamun AA (2015). Longitudinal impact of sleep on overweight and obesity in children and adolescents: a systematic review and bias-adjusted meta-analysis. Obes Rev.

[17] Magee CA, Caputi P, Iverson DC (2013). Patterns of health behaviours predict obesity in Australian children. J Paediatr Child Health.

[18] Saldanha-Gomes C, Marbac M, Sedki M, Cornet M, Plancoulaine S, Charles MA (2020). Clusters of diet, physical activity, television exposure and sleep habits and their association with adiposity in preschool children: the EDEN mother-child cohort. Int J Behav Nutr Phys Act.

[19] Glanz K, Rimer BK, Viswanath K (2015). Health behavior: theory, research, and practice.

[20] Sallis JF, Cervero RB, Ascher W, Henderson KA, Kraft MK, Kerr J (2006). An ecological approach to creating active living communities. Annu Rev Public Health.

[21] Stokols D (1992). Establishing and maintaining healthy environments: toward a social ecology of health promotion. Am Psychol.

[22] Van Koperen TM, Jebb SA, Summerbell CD, Visscher TLS, Romon M, Borys JM (2013). Characterizing the EPODE logic model: unravelling the past and informing the future. Obes Rev.

[23] Lu C, Huang G, Corpeleijn E (2019). Environmental correlates of sedentary time and physical activity in preschool children living in a relatively rural setting in the Netherlands: a cross-sectional analysis of the GECKO Drenthe cohort. BMJ Open.

[24] Halleck Vega S, Elhorst JP (2015). The slx model. J Reg Sci.

[25] L’Abée C, Sauer PJJ, Damen M, Rake J-P, Cats H, Stolk RP (2008). Cohort profile: the GECKO Drenthe study, overweight programming during early childhood. Int J Epidemiol.

[26] Centraal Bureau voor de Statistiek (CBS). StatLine: regionale kerncijfers Nederland. 2021.

[27] Centraal Bureau voor de Statistiek (CBS). StatLine: gezondheidsmonitor; bevolking 19 jaar of ouder, regio, 2016. 2018.

[28] Dutman AE, Stafleu A, Kruizinga A, Brants HA, Westerterp KR, Kistemaker C (2011). Validation of an FFQ and options for data processing using the doubly labelled water method in children. Public Health Nutr.

[29] Black AE (2000). Critical evaluation of energy intake using the Goldberg cut-off for energy intake:basal metabolic rate. A practical guide to its calculation, use and limitations. Int J Obes Relat Metab Disord.

[30] Schofield WN (1985). Predicting basal metabolic rate, new standards and review of previous work. Hum Nutr Clin Nutr.

[31] Vinke PC, Luitjens MHHS, Blijleven KA, Navis G, Kromhout D, Corpeleijn E (2020). Nutrition beyond the first 1000 days: diet quality and 7-year change in BMI and overweight in 3-year old children from the Dutch GECKO Drenthe birth cohort. J Dev Orig Health Dis.

[32] Vinke P, Corpeleijn E, Dekker L, Jacobs D, Navis G, Kromhout D (2018). Development of the Food-Based Lifelines Diet Score (LLDS) and its application in 129,369 lifelines participants. Eur J Clin Nutr.

[33] Choi L, Ward SC, Schnelle JF, Buchowski MS (2012). Assessment of wear/nonwear time classification algorithms for triaxial accelerometer. Med Sci Sports Exerc.

[34] Wiersma R, Lu C, Hartman E, Corpeleijn E (2019). Physical activity around the clock: objectively measured activity patterns in young children of the GECKO Drenthe cohort. BMC Public Health.

[35] Butte NF, Wong WW, Lee JS, Adolph AL, Puyau MR, Zakeri IF (2014). Prediction of energy expenditure and physical activity in preschoolers. Med Sci Sports Exerc.

[36] Fredriks AM, Van Buuren S, Burgmeijer RJ, Meulmeester JF, Beuker RJ, Brugman E (2000). Continuing positive secular growth change in The Netherlands 1955–1997. Pediatr Res.

[37] Cole TJ, Lobstein T (2012). Extended international (IOTF) body mass index cut-offs for thinness, overweight and obesity. Pediatr Obes.

[38] Pizzi C, Richiardi M, Charles MA, Heude B, Lanoe JL, Lioret S (2020). Measuring child socio-economic position in birth cohort research: the development of a novel standardized household income indicator. Int J Environ Res Public Health.

[39] Ministry for Internal Affairs. Catalogus BAG 2018 (BAG catalogue 2018). The Hague. 2018.

[40] van Buuren S, Groothuis-Oudshoorn K (2011). mice : multivariate imputation by chained equations in *R*. J Stat Softw.

[41] van Ginkel JR, Kroonenberg PM (2014). Using generalized procrustes analysis for multiple imputation in principal component analysis. J Classif.

[42] Gollini I, Lu B, Charlton M, Brunsdon C, Harris P (2015). GWmodel : an *R* package for exploring spatial heterogeneity using geographically weighted models. J Stat Softw.

[43] Lu B, Harris P, Charlton M, Brunsdon C (2014). The GWmodel R package: further topics for exploring spatial heterogeneity using geographically weighted models. Geo-spatial Inf Sci.

[44] Bivand RS, Wong DWS (2018). Comparing implementations of global and local indicators of spatial association. TEST.

[45] Bivand R, Pebesma EJ, Gómez-Rubio V (2013). Applied spatial data analysis with R.

[46] Bivand R, Hauke J, Kossowski T (2013). Computing the Jacobian in Gaussian spatial autoregressive models: an illustrated comparison of available methods. Geogr Anal.

[47] Gubbels JS, Kremers SPJ, Stafleu A, Goldbohm RA, de Vries NK, Thijs C (2012). Clustering of energy balance-related behaviors in 5-year-old children: lifestyle patterns and their longitudinal association with weight status development in early childhood. Int J Behav Nutr Phys Act.

[48] Wiersma R, Haverkamp B, van Beek J, Riemersma A, Boezen M, Smidt N (2019). Unravelling the association between accelerometer-derived physical activity and adiposity among preschool children: a systematic review and meta-analyses. Obes Rev.

[49] Bel-Serrat S, Ojeda-Rodríguez A, Heinen MM, Buoncristiano M, Abdrakhmanova S, Duleva V (2019). Clustering of multiple energy balance-related behaviors in school children and its association with overweight and obesity—WHO european childhood obesity surveillance initiative (COSI 2015–2017). Nutrients.

[50] Congdon P (2019). Obesity and urban environments. Int J Environ Res Public Health.

[51] Rijnks RH, Strijker D (2013). Spatial effects on the image and identity of a rural area. J Environ Psychol.

[52] Dekker LH, Rijnks RH, Strijker D, Navis GJ (2017). A spatial analysis of dietary patterns in a large representative population in the north of The Netherlands—the lifelines cohort study. Int J Behav Nutr Phys Act.

[53] van de Kassteele J, Zwakhals L, Breugelmans O, Ameling C, van den Brink C (2017). Estimating the prevalence of 26 health-related indicators at neighbourhood level in The Netherlands using structured additive regression. Int J Health Geogr.

[54] Cai Q, Dekker LH, Bakker SJL, de Borst MH, Navis GJ (2019). Intraregional differences in renal function in the northern Netherlands: the lifelines cohort study. PLoS ONE.

[55] Kamel Boulos MN, Koh K (2021). Smart city lifestyle sensing, big data, geo-analytics and intelligence for smarter public health decision-making in overweight, obesity and type 2 diabetes prevention: the research we should be doing. Int J Health Geogr.

[56] Christopher Prener TG, Biscale AZ. Tools and palettes for bivariate thematic mapping [R package biscale version 0.2.0]. 2020. https://cran.r-project.org/web/packages/biscale/index.html. Accessed 15 Jun 2021.

