# Supplementary material for: Regional variation in lifestyle patterns and BMI in young children: the GECKO Drenthe cohort
Source: Int J Health Geogr. 2022 Jul 1;21:7. doi: 10.1186/s12942-022-00302-7 (PMC9250228; doi:10.1186/s12942-022-00302-7)
Supplement: Supplementary file 1 — Additional file 1. Detailed description of the spatial analysis. [file 12942_2022_302_MOESM1_ESM.docx]

**Additional file 1.** Detailed description of the spatial analysis

For the spatial analysis we started with a general nesting spatial model (for a more detailed description of all available models see Vega and Elhorst^21^). The general nesting spatial model takes the form

$$Y=\rho WY + \alpha+ X\beta+ WX\theta+ u,$$

$$u = \lambda Wu + \varepsilon$$

 where the terms *Y*, $\alpha,$ $X\beta,$and $\varepsilon$ are the conventional terms for the dependent variable, intercept, independent variables and error term. The *W* represents a spatial weights matrix which specifies the linkages between neighbours. In its simplest form, the *W* takes the value 1 if two observations are neighbours, and 0 if they are not. For this study, the spatial weights matrix was constructed using a k-nearest neighbours, with k set at 10, to ensure an equal number of neighbours were included in the calculation of the lagged variable for each case. The percentage of non-zero weights was 0.558, and the median link distance 0.438 kilometres (min=0km, Q1=0.243km, Q3=0.895km, max=6.768km). The term $\rho WY$ measures the spatial autocorrelation in the dependent term. A significant coefficient for $\rho$ would indicate that higher local BMI is associated with higher individual BMI. $WX\theta$ measures the spatial spillover effect of neighbouring independent variables on the individual. Finally, $\lambda Wu$ measures the spatial autocorrelation of the error term. Unobserved processes, consigned to the error term, may display spatial autocorrelation. Ignoring this leads to inefficient estimation and larger standard errors for the coefficients. If all spatial coefficients are constrained to 0, the model defaults back to a standard OLS. We follow convention by first estimating the general nesting spatial model, including all spatial coefficients, and subsequently constrain insignificant spatial coefficients to 0. From the general nesting spatial we fit a spatial autoregressive combined ($\theta=0$) model, a spatial durbin error ($\rho=0$) model and spatial error ($\theta=0 and \rho=0$) model. All regression models were performed using *spatialreg* in each imputed dataset separately, and combined using Rubin’s rules ^38–40^. As sensitivity analyses, we also performed the analyses with one pattern at a time and performed the analyses on the subset of complete cases.
