# Supplementary material for: Regional variation in lifestyle patterns and BMI in young children: the GECKO Drenthe cohort
Source: Int J Health Geogr. 2022 Jul 1;21:7. doi: 10.1186/s12942-022-00302-7 (PMC9250228; doi:10.1186/s12942-022-00302-7)
Supplement: Supplementary file 2 — Additional file 2. Description of missing data. [file 12942_2022_302_MOESM2_ESM.docx]

| **Additional file 2.** Description of missing data | | | | | |
| --- | --- | --- | --- | --- | --- |
|  | | | *Age of measurement* | *Information missing* | |
|  | | | *Median [95%CI]* | *N* | *%* |
| *Lifestyle factor* | | | | | |
|  | Diet | | 3.1 [2.9 – 3.4] | 712 | 39.2% |
|  | Outdoor play | | 3.9 [3.8 – 4.2] | 991 | 54.5% |
|  | Screen time | | 3.9 [3.8 – 4.2] | 992 | 54.6% |
|  | Sleep | | 6.0 [5.5 – 6.6] | 446 | 24.5% |
|  | Sedentary time and physical activity | | 5.7 [4.4 – 7.0] | 807 | 44.4% |
| *Adiposity* | | | | | |
|  | zBMI 5 years | | 5.8 [5.3 – 6.4] | 150 | 8.3% |
| *Socio-economic status* | | | | | |
|  | | Equivalized household income indicator | Birth | 104 | 5.7% |
|  | | Maternal education level | Birth | 39 | 2.1% |
|  | |  | | | |
