# Supplementary material for: Regional variation in lifestyle patterns and BMI in young children: the GECKO Drenthe cohort
Source: Int J Health Geogr. 2022 Jul 1;21:7. doi: 10.1186/s12942-022-00302-7 (PMC9250228; doi:10.1186/s12942-022-00302-7)
Supplement: Supplementary file 3 — Additional file 3. Additional regression models for the prospective associations of the lifestyle patterns with standardized body mass index at 10–11 years. [file 12942_2022_302_MOESM3_ESM.docx]

**Additional file 3.** Additional regression models for the prospective associations of the lifestyle patterns with standardized body mass index at 10-11 years.

| **Table I.** Unadjusted regression model for the prospective associations of the lifestyle patterns with standardized body mass index at 10-11 years | | | | | | |
| --- | --- | --- | --- | --- | --- | --- |
|  | Unadjusted model | | Model 1^a^ | | Model 2^b^ | |
|  | B | 95% CI | B | 95% CI | B | 95% CI |
| *Lifestyle patterns* |  |  |  |  |  |  |
| ‘High activity’ | 0.013 | (-0.03; 0.05) | 0.005 | (-0.02; 0.03) | 0.002 | (-0.03; 0.03) |
| ‘Low screen time, high sleep and healthy diet’ | **-0.142** | **(-0.19; -0.09)** | **-0.076** | **(-0.11; -0.04)** | **-0.072** | **(-0.11; -0.04)** |
| ‘High outdoor play’ | 0.047 | (-0.02; 0.12) | 0.036 | (-0.01; 0.09) | 0.031 | (-0.02; 0.08) |
| *Socio-economic status* |  |  |  |  |  |  |
| Equivalized Household Income Indicator | **-** | **-** | **-** | **-** | **-0.054** | **(-0.09; -0.02)** |
| *Additional variables* |  |  |  |  |  |  |
| Age BMI measurement | **-** | **-** | 0.004 | (-0.07; 0.08) | -0.010 | (-0.09; 0.07) |
| Sex | **-** | **-** | -0.041 | (-0.11; 0.03) | -0.046 | (-0.12; 0.02) |
| Weartime | **-** | **-** | 0 | (-0.001; 0.002) | 0 | (-0.001; 0.002) |
| Energy intake | **-** | **-** | 0 | (0.000; 0.000) | 0 | (0.000; 0.000) |
| Smoking during pregnancy | **-** | **-** | -0.001 | (-0.10; 0.10) | -0.017 | (-0.12; 0.08) |
| zBMI 5-6 yrs. | **-** | **-** | **0.944** | **(0.90; 0.98)** | **0.940** | **(0.90; 0.98)** |
| *^a^ Model 1: Analyses were adjusted for age, sex, weartime, energy intake, smoking during pregnancy and standardized body mass index (zBMI) at 5-6 years.*  *^b^ Model 2: Model 1 + Equivalized Household Income Indicator.* | | | | | | |

| **Table II.** Spatial autoregressive combined and spatial durbin error regression models for the prospective associations of the lifestyle patterns with standardized body mass index at 10-11 years | | | | |
| --- | --- | --- | --- | --- |
|  | Spatial autoregressive combined model | | spatial durbin error model^a^ | |
|  | B | 95% CI | B | 95% CI |
| *Lifestyle patterns* |  |  |  |  |
| ‘High activity’ | 0.003 | (-0.03; 0.03) | 0.002 | (-0.03; 0.03) |
| ‘Low screen time, high sleep and healthy diet’ | **-0.071** | **(-0.11; -0.033)** | **-0.071** | **(-0.11; -0.03)** |
| ‘High outdoor play’ | 0.031 | (-0.02; 0.08) | 0.031 | (-0.02; 0.08) |
| *Socio-economic status* |  |  |  |  |
| Equivalized Household Income Indicator | **-0.052** | **(-0.09; -0.02)** | **-0.051** | **(-0.09; -0.02)** |
| *Spatial components* |  |  |  |  |
| Rho (spatial term for zBMI 10-11 years) | 0.060 | (-0.06; 0.18) |  |  |
| lag_SES |  |  | -0.055 | (-0.156; 0.05) |
| lag_’high activity’ |  |  | -0.023 | (-0.11; 0.07) |
| lag_’low screen time, high sleep and healthy diet’ |  |  | -0.026 | (-0.13; 0.08) |
| lag_’high outdoor play’ |  |  | -0.03 | (-0.19; 0.13) |
| Lambda (spatial term for error) | 0.061 | (-0.11; 0.23) | **0.108** | **(0.00; 0.22)** |
| *Analyses were adjusted for age, sex, weartime, energy intake, smoking during pregnancy and standardized body mass index (zBMI) at 5-6 years.*  *^a^ Additionally adjusted with a spatial term for zBMI at 5-6 years.* | | | | |
